# Treatment of Distal Radius Fractures with Bridging External Fixator with Optional Percutaneous K-Wires: What Are the Right Indications for Patient Age, Gender, Dominant Limb and Injury Pattern?

**DOI:** 10.3390/jpm12091532

**Published:** 2022-09-18

**Authors:** Carlo Biz, Mariachiara Cerchiaro, Elisa Belluzzi, Elena Bortolato, Alessandro Rossin, Antonio Berizzi, Pietro Ruggieri

**Affiliations:** 1 Orthopedics and Orthopedic Oncology, Department of Surgery, Oncology and Gastroenterology, University of Padova, Via Giustiniani 3, 35128 Padova, Italy; 2 Musculoskeletal Pathology and Oncology Laboratory, Department of Surgery, Oncology and Gastroenterology, University of Padova, Via Giustiniani 3, 35128 Padova, Italy; 3 Department of Statistical Sciences, University of Padova, 35121 Padova, Italy

**Keywords:** distal radius fracture, wrist injury, bridging external fixator, external fixator, K-wires, QuickDASH, PRWHE score

## Abstract

The aim of this retrospective study was to evaluate the medium-term clinical and functional outcomes of patients with closed, displaced, and unstable, simple or complex, intra- and extra-articular distal radius fractures (DRFs) treated with a bridging external fixator (BEF) and optional K-wires (KWs). AO classification was used to differentiate the injuries radiographically. Clinical-functional outcomes were evaluated using the Patient-Rated Wrist and Hand Evaluation Score (PRWHE Score) and the Quick Disabilities of the Arm Shoulder and Hand Score (QuickDASH). A total of 269 dorsally displaced fractures of 202 female (75%) and 67 male subjects (25%) were included, with a mean follow-up of 58.0 months. Seventy-five patients (28%) were treated by additional KWs. No differences were found comparing the two groups of patients (BEF vs. BEF + KWs) regarding age, sex, and fracture side (dominant vs. non-dominant). PRWHE and QuickDASH scores were lower in the BEF + KWs group compared to the BEF group (*p* < 0.0001 and *p* = 0.0007, respectively). Thus, patients treated with KWs had a better clinical outcome. Beta multivariate regression analysis confirmed that patients of the BEF + KWs group exhibited a better PRWHE score but not a better QuickDASH score. Patients treated by the BEF + KWs with the fracture on the dominant site were characterised by better clinical outcomes. Older patients had a better PRWHE score independently from the treatment. Our findings suggest that the use of BEF for DRFs with optional KWs can be indicated in both young and elderly patients of any gender, independent of limb side and fracture pattern. As the best functional results were achieved in the elderly when KWs were added, the combination of BEF and KWs seems to be mainly indicated for the treatment of DRF, also complex, in the elderly population.

## 1. Introduction

Distal radius fractures (DRFs) are one of the most common acute events in traumatology, accounting for 17% of all fractures and 1.5–2.5% of access to emergency departments [1]. These fractures are the first in frequency in the general population, followed by fractures of the finger phalanges (11.2%), the metacarpal bones (10.5%), and the proximal femur (8.9%) [2,3,4,5,6]. According to recent studies [7,8,9], 98.3% of distal forearm fractures have been reported as radius fractures, which may be associated with ulna fractures, and approximately 55% of DRFs are associated with ulnar styloid fractures.

Regarding the trauma mechanism, most fractures are caused by a fall on the hand with the wrist in extension. The pattern and severity of the DRF, as well as the concomitant injuries of the associated disco-ligament structures, depend on the position of the wrist at the moment of impact and the severity of bone fragility. Certainly, the amplitude of this angle influences the location of the fracture [1,10]. For these reasons, the DRFs present a peak in incidence among men under 30 and a peak in women over 60 with osteoporosis [10]. Fractures in young adult patients are usually caused by high-energy trauma, while low-energy dynamics are the most common in geriatric patients [5,11,12]. DRFs are rarely associated to ipsilateral elbow dislocation and instability [13,14].

Depending on the fracture pattern and the AO classification, the therapeutic indication differs [15]. Conservative treatment is preferred mostly for simple undisplaced extra-articular fractures, especially in older low-demand patients and patients who do not require a quick return to work [15,16,17]. Surgical treatment is necessary in cases of displaced or unstable extra-articular DRFs, simple or complex intra-articular fractures, or in the case of secondary loss of reduction of the fracture [18]. For these, different operative options are available: osteosynthesis with volar or dorsal plates and screws, external wrist fixation, percutaneous fixation with Kirschner wires (KWs), or combinations of the previous two methods. The choice of surgical treatment and the superiority of one method over the others are still debated in the community of orthopaedic surgeons [17,19,20,21].

Since 2008, external fixation (EF) has become a popular technique in treating unstable DRFs with satisfactory functional results. In this method, some KWs (1.8–2.0 mm) can be added and driven into distal fracture fragments at different angles for improving DRF reduction [22]. Several advantages have been described by using an EF: anatomical reduction of the fracture under fluoroscopic view; increase in reduction by ligamentotaxis, together with the ability to preserve the reduction until the break is healed; simple application of the hardware; minimum operative X-ray exposure; and the reduction of surgical operation time [23]. This type of fixation can be divided into bridging EF and non-bridging EF. In current orthopaedic practice, there are three main types of EF available for adults: F-wrist, Hoffman II Compact, and Pennig Dynamic Wrist Fixator [24].

Comminuted fracture patterns are often difficult to reduce and maintain the reduction over time. Hence, additional KWs are needed as a reduction tool, providing additional stability to the fracture site. To date, only a few studies have focused on the role of optional KWs in EF [12,21], specifically in relation to the bridging fixation technique, and their proper indications is still a source of controversy among orthopaedic surgeons.

The aim of the present study was to investigate and evaluate the medium-term clinical and functional outcomes of patients with closed, displaced, unstable, simple or complex, intra-articular and extra-articular DRFs treated with a bridging external fixator (BEF) and optional percutaneous KWs.

## 2. Materials and Methods

### 2.1. Patients

This study was designed as a retrospective, single-centre, comparative, clinical, and functional study, including a consecutive series of Caucasian patients affected by DRFs and treated at our level-I healthcare trauma centre from January 2014 to December 2019 using BEF (Citieffe ST.A.R.90 F4 wrist was the EF available at our institution) with or without additional KWs. At the pre-operative period, all DRFs analysed were classified radiographically according to the AO classification [25].

### 2.2. Ethics

All subjects participating in this medium-term follow-up study received a thorough explanation of the risks and benefits of inclusion and gave their written informed consent to participate in the study. The approval of the Local Ethics Committee of Padova was obtained (266n/AO/22). The currently reported retrospective cohort study was performed in accordance with the ethical standards of the 1964 Declaration of Helsinki as revised in 2013 and conducted ethically according to the most recent international standards [26].

### 2.3. Inclusion and Exclusion Criteria

The inclusion criteria were: (1) closed, displaced (fragments not in anatomical alignment with dorsal angulation >15–20 degrees and radial shortening >3 mm [27]) and unstable (comminuted or articular fractures and loss of position following manual reduction [28]) DRFs, extension fractures (Colles’s) and articular extension ones treated with BEF and optional KWs; (2) age over 18 at the time of surgery; (3) operation carried out within 72 h after the traumatic event; (4) isolated complete extra-articular or articular fracture of the distal radius; (5) patients who had complete medical records; and (6) patients with a history of physiokinesitherapy for rehabilitation after the removal of BEF.

The exclusion criteria were: (1) open, stable, undisplaced or incomplete DRFs, flexion fractures (Smith) and articular flexion ones; (2) patients who had undergone re-operation or those who had undergone internal osteosynthesis by EF; (3) patients with significant comorbidities (i.e., diabetes, rheumatological, oncological, neurological, or cognitive types, and systemic infection); (4) polytraumatic patients; and (5) patients with previous DRF who were not willing to cooperate with the treatment.

### 2.4. Surgical Percutaneous Techniques

**Figure 1:**
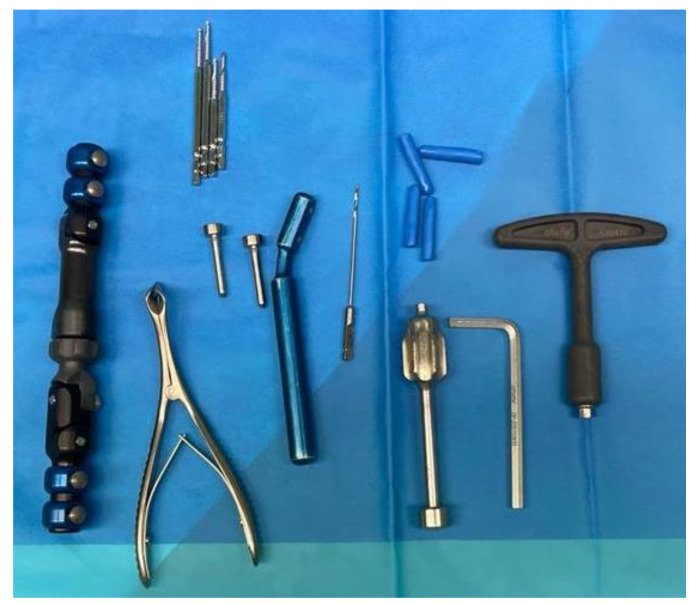
The complete kit of the Bridging External Fixator (BEF) for the wrist by Citieffe.

All operations were performed under brachial plexus regional anaesthesia at our institution by the same trauma team of two surgeons with the patient in supine position on the operating table and maintaining the affected forearm pronated (Figure 1).

**Figure 2:**
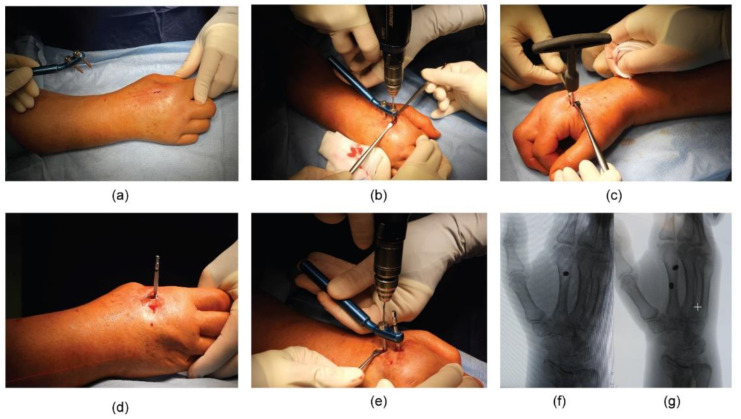
Intra-operative images showing: (**a**) dorsal skin incision at the base of the second metacarpal bone; (**b**–**d**) insertion of the first distal bicortical pin; (**e**) insertion of the second distal cortical pin using the proper tool to maintain the right distance between the pins; antero-posterior (AP) fluoroscopic control of (**f**) the first and (**g**) the second distal pin on the second metacarpal bone.

First, a dorsal incision and blunt dissection were performed at the base of the second metacarpal bone. Two threaded 3 mm bicortical metacarpal pins were introduced into the metacarpal bone at an angle of inclination between 30° and 45° with respect to the horizontal plane (Figure 2).

**Figure 3:**
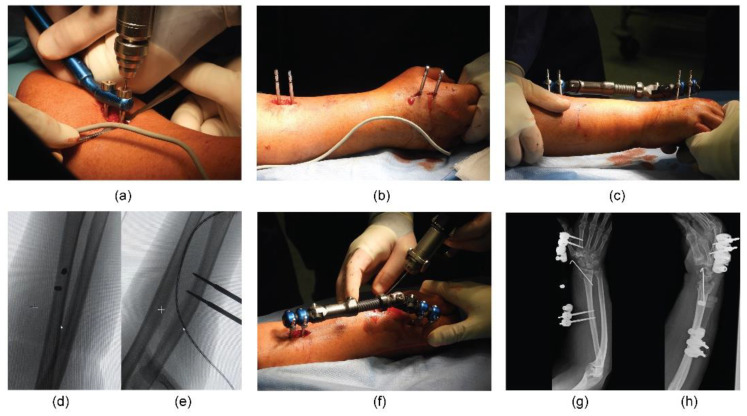
Intra-operative images showing: (**a**) insertion of the pins in the radial diaphysis between the middle third and the distal third of the forearm; (**b**) all pins positioned correctly; (**c**) final application of BEF; (**d**) antero-posterior (AP) and (**e**) latero-lateral (LL) fluoroscopic views of the proximal pins; (**f**) insertion of the KW at the radial styloid level. Post-operative radiographic images: (**g**) AP and (**h**) LL views showing proper reduction and stabilisation of the DRF (AO: 23-C2).

Second, a longitudinal dorsal incision at the radial border between the middle third and the distal third of the forearm (about 10 cm proximal to the wrist) was performed. After divarication of the subcutis protecting the cutaneous sensory branch of the radial nerve, the bone plane was reached to implant the two bicortical radial pins, respecting the same inclination of 30–45° as the horizontal plane of the forearm. Positioning the BEF body by fixing it to the pin, the fracture reduction was obtained by external manoeuvres under fluoroscopic control, maintaining the wrist in flexion position to stabilise the fracture. Under fluoroscopic control, a percutaneous KW (1.6 mm) was inserted in addition to BEF, from the radial styloid or the dorsum of the radius across the fracture fragments at the surgeon’s discretion. The KWs applied were left protruding about 1 cm from the skin and bent so as not to penetrate the soft tissues during the following weeks. After fixation, fluoroscopy was used to check the restoration of volar tilt, radial inclination, ulnar variance, radial height, palmar tilt, articular joint congruency, and external fixator placement (Figure 3).

At the end of the surgery, bandages were placed around the pin of the BEF. The external fixator devices and the optional KWs were removed four to five weeks from surgery as an outpatient procedure after radiographic control. The exact timing varied depending on the pin site stability and radiographic evidence of bone fusion or temporary callus bone bridging formation [12,29].

### 2.5. Post-Operative Protocol of Both Procedures

All patients followed the same post-operative protocol and were followed in the same standardised manner by the senior authors. Active and passive finger mobility was immediately granted. Antero-posterior and lateral X-rays of wrist were taken before the patients were discharged. We recommended an anti-oedemigen therapy (Leucoselect, Lymphaselect, and bromelain: 1 cp/day) for 30 days, starting from the day of the surgery; an analgesic therapy for two weeks with etoricoxib (90 mg, 1 cp/day) in the morning; and if pain persisted, paracetamol/phosphate codeine (1 g, max x3/day) was prescribed. The dressing of the surgical sutures took place seven and fourteen days after surgery, and the removal of sutures on the fourteenth post-operative day. During each scheduled visit, medication at the pin and KW sites was performed until metalwork removal. Post-operative and one- to four-week radiographic checks were performed with subsequent outpatient removal of the body and external wrist fixator pins, including KWs when presented. The remaining wounds at the sites of the pins and KWs were allowed to heal spontaneously.

**Figure 4:**
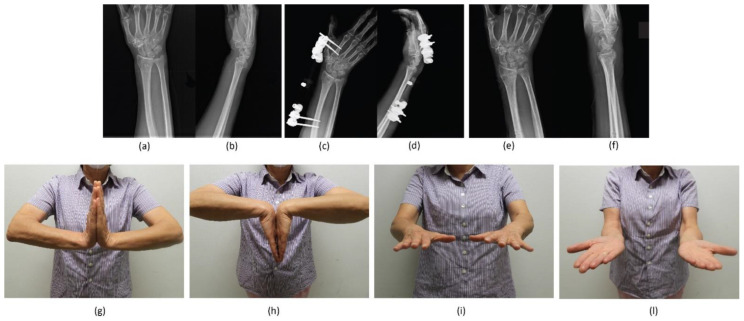
A 69-year-old female patient treated with BEF for an AO 23-B3 DRF. Antero-posterior (AP) and latero-lateral (LL) radiographic images at (**a**,**b**) pre-operative period; (**c**,**d**) immediate post-operative period; (**e**,**f**) at 2-month follow-up. Clinical-functional images showing: (**g**) extension, (**h**) flexion, (**i**) pronation, and (**l**) supination of the operated wrist at last follow-up.

**Figure 5:**
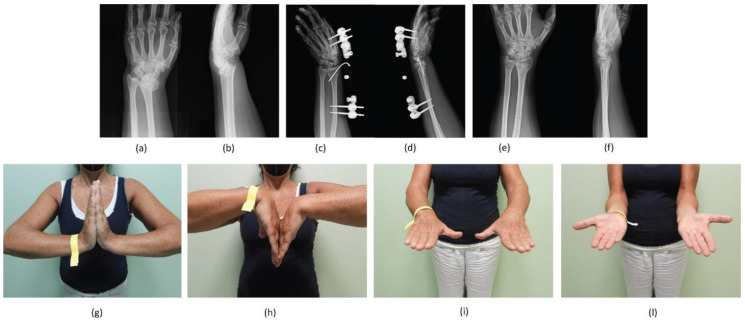
A 58-year-old female patient treated with BEF and an additional KW for an AO 23-C2 DRF. Antero-posterior (AP) and (**b**) latero-lateral (LL) radiographic images at (**a**,**b**) pre-operative period; (**c**,**d**) immediate post-operative period; (**e**,**f**) at 2-month follow-up. Clinical-functional images showing: (**g**) extension, (**h**) flexion, (**i**) pronation, and (**l**) supination of the operated wrist at last follow-up.

The physiokinesitherapy process started with the removal of the fixator for an average duration of about three weeks. Active and passive mobilisation was started immediately; resistance activity was granted starting one week after the removal of the fixator. The daily and work activities were gradually resumed, reaching the absence of limitations twelve weeks after the surgery [12] (Figure 4 and Figure 5).

### 2.6. Patient Assessment

Patient characteristics (gender and age at trauma), fracture classification, and dominant side were collected at the baseline. At last follow-up, participants were invited to fill out two different questionnaires for the evaluation of wrist function: the Patient-Rated Wrist Hand Evaluation (PRWHE) and the Quick Disability of the Arm, Shoulder, and Hand (QuickDASH) [30,31]. Both questionnaires are scored from 0 to 100, with higher scores indicating greater disability.

Post-operative complications were recorded and divided between minor and major. Minor complications included wound complications, pain, swelling, and weakness. Major complications included deep infection, chronic infection, non-union, malunion, median nerve-related complaints, and complex regional pain syndrome (CRPS).

According to the surgical treatment, the patients were divided into two groups:

(1) BEF group;
(2) BEF + KWs group.

### 2.7. Statistical Analysis

Normality of data distribution was verified by conducting the Shapiro–Wilk test. Continuous variables were expressed as mean and standard deviation, while for categorical variables, absolute frequencies and percentage were reported when appropriate. Univariate (Student’s *t*-test for unpaired, independent samples, chi-squared test) and multivariate analyses were conducted to assess eventual differences between the two surgical techniques. To understand the effect of the treatment, possible confounders (i.e., age, hand dominance, and sex) were taken into account. It was also noted that the treatment assignment could be influenced by pre-surgery status, which may affect the clinical outcome. Thus, to eliminate the non-randomness of the assignment and to be able to identify the direct effect of treatment on the clinical outcome and that of the covariates, the propensity score (pp) was used. This score measures the treatment dependence on pre-surgery status; hence, conditional on the propensity, the treatment is independent of the pre-surgery status.

The pp was estimated using logistic regression with bias correction [32] and used in the second stage model that also included the treatment and the confounder variables.

For properly modelling the outcome scores, a beta regression model was used that was found to be more suitable in terms of distributional assumptions based on the data. Bias correction techniques implemented in the R package betareg and brglm2 [33] ensured more accurate estimates of standard errors compared to standard maximum likelihoods.

For all analyses, values with *p* values less than or equal to 0.05 were considered statistically significant. All statistical analyses were performed using R [34].

## 3. Results

### 3.1. Patient Data

**Figure 6:**
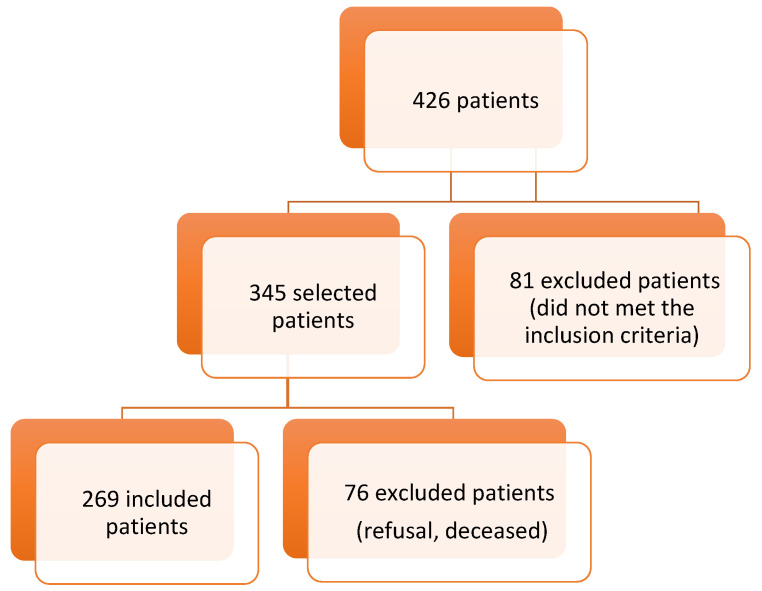
Flowchart of patient selection.

During a six-year period of enrolment in our institute, 426 patients were treated for dorsally displaced and unstable DRFs with BEF of the wrist. A total of 81 patients were excluded according to the inclusion/exclusion criteria, and a final cohort of 345 patients met the inclusion criteria of this study. Of those, 269 patients were included and completed the follow-up, while 76 patients were excluded (died, were not found, or refused to participate to the study). Therefore, a final cohort of 269 patients was enrolled (Figure 6). All patients were evaluated after an average follow-up of 58 months after surgery (range: 24–87 months).

The mean age of the 269 study participants was 65.54 years at the time of surgery (range: 18–94 years); 202 patients were female (75%) and 67 were male (25%). Fractures of the dominant side occurred in 106 patients (39.4%), while the remaining fractures were on the non-dominant side (163 patients, 60.6%). The mean PHRWE was 12.18 ± 15.75 and mean QuickDASH was 13.13 ± 15.86 of the overall cohort at the follow-up. Fractures were classified using the AO classification system (Table 1).

Considering the overall cohort, no differences between PRWHE and AO classification (*p* = 0.40) or gender (*p* = 0.35) were observed. Likewise, no differences between QuickDASH and AO classification (*p* = 0.43) or gender (*p* = 0.21) were found. A weak negative correlation was found between clinical scores and age (PRWHE: r = −0.21; *p* < 0.001 and QuickDASH: r = −0.22; *p* < 0.001). Of the 266 cases, 75 (28%) were treated by associating KWs to the BEF. No differences were found comparing the two groups of patients (BEF vs. BEF + KWs) regarding age, sex, and fracture side (dominant vs. non-dominant). PRWHE and QuickDASH scores were lower in BEF + KWs group compared to BEF, indicating that patients with KWs have a better clinical outcome (*p* < 0.0001 and *p* = 0.0007, respectively) (Table 2).

### 3.2. Complications

Minor complications were recorded in 70 patients (26%). Discomfort due to the presence of the BEF was reported by 23 patients. Twenty patients had superficial pin infections, which were successfully treated with oral antibiotics. Complex regional pain syndrome (CRPS) occurred in six patients. Radial nerve injury was found in four patients. Radial shortening was recorded in five patients. Tendon irritation occurred in 12 patients.

### 3.3. Multivariate Analysis

With the beta multivariate regression analysis, it was confirmed that patients of the BEF + KWs groups exhibited a better PRWHE score; however, no influence was reported with QuickDASH. Patients of the BEF + KWs with the fracture on the dominant site were characterised by a better clinical outcome (both PRWHE and QuickDASH). Older patients had a better PRWHE score independently from the treatment. No influence was observed regarding fracture type and fracture type associated with treatment type (Table 3 and Table 4).

In order to interpret the effect sizes estimated by the model, some examples of predicted scores could be compared that differ for a change of variable associated to a significant coefficient in the model. Taking as a reference group the one with median characteristics for quantitative variables (propensity score equal to 0.27, age 68), fracture of type A2, no KWs, and non-dominant side, the estimated scores were 13.29 for PRWHE and 15.31 for QuickDASH. For the group with the same characteristics but dominant side, the scores became 16.79 and 20.48, respectively. Changing the treatment group from BEF to BEF + K-wires, the scores within the reference group dropped from 13.29 to 9.44 (PRWHE) and from 15.31 to 12.34 (QuickDASH) for the non-dominant side, and from 16.79 to 8.34 (PRWHE) and from 20.48 to 11.16 (QuickDASH) for the dominant side.

Regarding age, there is a progressive decrease of the scores as the age of patients increases, keeping all of the other characteristics fixed. The predicted range is between 10.98 for 20-year-old individuals and 26.80 for 80-year-old individuals (PRWHE) and from 12.34 for 20-years-olds to 32.52 for 80-year-olds (QuickDASH).

## 4. Discussion

DRFs are common orthopaedic injuries, occurring within 3 cm of the distal part of the radius, with a bimodal age distribution (a peak in incidence among men under 30 and a peak in women over 60 with osteoporosis). The two main trauma mechanisms are high-energy trauma and low-energy falls, respectively [5,7].

Management of DRFs includes closed reduction and casting, closed reduction and percutaneous fixation, and open reduction internal fixation with volar or dorsal plating [35]. Immobilisation with wrist casts is the conservative solution reserved for simple displaced or minimally displaced extra-articular fractures, especially if the patient does not require a quick return to work [16,36,37]. About 35% of total DRFs require surgery. Unstable extra-articular and intra-articular fractures of the distal epiphysis of the radius usually require surgical treatment [36,38]. For these injuries, however, there is still no agreement on what the optimal treatment is in relation to age, gender, dominant limb, and fracture pattern.

In our study, we compared the scores of two questionnaires for the evaluation of functional outcomes in patients with DRFs treated with a BEF and optional KWs divided into categories on the basis of age, type of treatment, pattern fracture, gender, and limb dominance. Only closed, displaced, and unstable extension fractures (Colles’s) and articular extension fractures were included, because flexion fractures (Smith) and articular flexion fractures were treated by plate osteosynthesis according to our institutional wrist trauma protocol. The most important finding of our analysis was that no differences between PRWHE and AO classification (*p* = 0.40) or gender (*p* = 0.35) were observed in our overall cohort at the mean follow-up of 58 months. Likewise, no differences between QuickDASH and AO classification (*p* = 0.43) or gender (*p* = 0.21) were found.

Although the comparison of EF versus volar plates and screws (VLP) in unstable DRF was not the objective of this study, a meta-analysis comparing both techniques concluded that cases treated with a VLP could obtain better functional outcomes [39]. In contrast, a recent meta-analysis and systematic review showed that patients treated with VLP had a lower DASH score and VAS score, and no significant differences in radiographic outcomes were observed even if VLP had a lower complication rate than that of EF [29]. Another study reported that VLP fixation resulted in faster recovery of function compared to EF, but no functional advantage was demonstrated at two years’ short follow-up [40]. Furthermore, patients undergoing DRF surgery with open reduction and internal fixation (ORIF) have a higher risk of wound infection and tendonitis [41].

Hence, the results from the literature suggest that surgery yields statistically but not clinically better functional outcomes at one-year follow-up [42]. Treatment with VLP might benefit patients who have the need to gain the previous level of activity in a short period of time. However, it deserves mention that the current literature does not provide an actual cut-off for age, fracture malalignment, or other specific factors.

Regarding the population age, some authors have underlined a variety of differences in demographic factors, considering the EF more commonly indicated in younger, male patients who are more likely to have higher energy trauma and more significant distal radius comminution [43]. On the contrary, in our cohort, a weak negative correlation was found between clinical scores and age (PRWHE: r = −0.21; *p* < 0.001 and QuickDASH: r = −0.22; *p* < 0.001). Specifically, the difference in terms of functional results between EF in the elderly and in the younger patients was not statistically significant, although young people require a greater degree of functionality than the elderly and a better possible reduction in fracture, which is not always anatomically obtainable by BEF. Furthermore, the general evaluation of the results between AO 23A and AO 23C fracture pattern did not show statistical significance among young and elderly patients. These findings can be justified by the fact that the functional-clinical outcomes can be perceived subjectively in different ways for the different injury patterns by the two populations.

Certainly, BEF is not always able to guarantee the alignment of the fracture fragments and the stability of the reduction, especially in cases of articular or metaphyseal involvement or severe periarticular injury [44]. For these reasons, in some cases, we preferred associated percutaneous KWs, which were useful to reduce and stabilise the fractures during surgery, and they contributed to achieving good functional outcomes at medium-term follow-up, which are shown in the BEF + KWs group. Rectenwald et al. [45] also demonstrated how the association of EF and KWs not only allows improvement of stability but also maintains the reduction obtained with simple EF until bone callus formation.

To date, only a few studies have focused on the role of additional KWs in EF [12]. Among the 269 cases included in our study, 75 (28%) were treated by associating percutaneous synthesis with KWs to the BEF. No differences were found comparing the two groups of patients (BEF vs. BEF + KWs) regarding age, sex, and fracture side (dominant vs. non-dominant), and all patients of both groups were able to resume normal daily life activities after the operation. However, PRWHE and QuickDASH scores were lower in the BEF + KWs groups compared to EF (*p* < 0.0001 and *p* = 0.0007, respectively), indicating that patients with KWs had a better clinical outcome. Patients of the BEF group with a fracture on the dominant site had a worse clinical outcome (higher scores) than those with the fracture on the non-dominant side. The opposite occurred in the BEF + KWs group: patients with the fracture on the dominant side had better clinical outcomes. In other words, the predicted scores for patients with the dominant side injured are better (lower) for the patients treated with BEF + KWs than with the BEF alone. In the group with the non-dominant side injured, the clinical outcome is still better for those treated with BEF + KWs with respect to those treated with BEF only, even if the difference is less evident. It should be noted that the scores obtained are very good. Importantly, beta multivariate regression analysis confirmed that the BEF + KWs group exhibited a better PRWHE score; however, no influence was reported with QuickDASH. However, patients of the BEF + KWs with the fracture on the dominant site were characterised by a better clinical outcome (both PRWHE and QuickDASH). Older patients had better PRWHE scores independently of the treatment, probably because of low demand. No influence was observed regarding fracture type and fracture type associated with treatment type. Our clinical functional results are in line with those of another scientific report: Fu et al. [46] demonstrated how the combination of BEF and KWs leads to better clinical-functional results than simple BEF.

In the literature, there is agreement in the definition of what is the least clinically relevant difference between mean functional scores: according to the meta-analyses of Li-hai et al. (2015) [47] and Walenkamp et al. [48] and the study of Gummesson et al. [49], the minimal score difference describing a clinically significant difference after two upper limb surgical treatments is a mean difference of 10 points. In our study, we recorded significant differences between means greater than 10 points (or approximately 10 points), in agreement with the literature.

Wrist EF has many advantages in the treatment of DRFs. First, it scarcely affects the blood supply around the fracture ends, which is conducive to recanalisation of bone vessels and creates a good fracture-healing environment. Second, non-cross-joint fixation allows normal movement of the wrist, diminishes stiffness, stimulates cartilage repair, decreases osteopenia of the distal fragment and reduces fear among patients [41].

In our groups, fractures of the dominant side occurred in 106 patients (39.4%), while the remaining fractures were on the non-dominant side (163 patients, 60.6%). The analysis of the data relative to the dominant limb is interesting as it did not show major functional deficits in daily activities with respect to the non-dominant limb between the two procedure groups. Hence, good results were recorded for the dominant limb independent of the procedure used. Our results relating to the proper use of BEF, with optional KWs, in DRFs in relation to patient age, gender, dominant limb, and injury pattern do not find comparable precedents in the literature.

Given the known epidemiological difference in the prevalence of DRFs, no significant differences were found in the outcomes of patients of different genders, which is simple evidence that gender-specific factor-related activities are not involved in the subjectivity of filling out the outcome questionnaires. These results agree with the results of other scientific reports presented in literature: the study by Lee et al. [50] did not identify statistical significance for the gender variable in terms of post-operative functional results; Synn et al. [17] found no influence of patient gender.

In the literature, EF use for the treatment of DRF is associated with a 24% to 62% complication rate, most of which includes superficial pin site infection, malunion, and loss of radiocarpal and digital motion [51,52,53,54,55,56]. Specifically, Weber and Szabo reported a 62% complication rate associated with EF, most commonly loose pins, pin tract infection, and malreduction [55].

Normally, pin tract infections range between 0 and 27% [55,57,58,59]. However, Anderson et al. reported 37.5% pin tract infection; all infections were resolved with antibiotics [52]. Raskin and Melone reported no pin tract infections in their study [60]. They attribute this to their method of pin site care. Carpal tunnel syndrome (4.3% non-operative, 1.9% surgery) has been reported more commonly in non-operatively treated patients and in those treated by VLP [42]. In our study, the minor complications registered (superficial pin infection, discomfort from external hardware, and tendon irritation) were all resolved in a short time, and none affected the clinical functional outcomes at last follow-up. For these reasons, as trauma surgeons, we think these must be considered part of the surgical procedure and the post-operative period rather than sequalae of the treatment method. On the contrary, the few major complications reported—carpal tunnel syndrome, CRPS, radial nerve injury, and radial shortening—impacted medium-term results. We did not find malunion or nonunion, contrary to what is reported in the study of Anderson at al. [52], where their incidence was surprisingly high (12.5%).

Over-distraction can cause increased pressure in the carpal tunnel, according to Gelberman et al. [61]. To avoid this, Hertel and Ballmer suggest first obtaining preliminary reduction with over-distraction and then stabilising the fracture with crossed KWs, followed by reduction of distraction to neutral length and position [62]. The incidence of superficial radial nerve irritation could be largely dependent on the surgeon’s technique of pin placement. Using an open technique, the superficial branch of the radial nerve can be protected.

Discomfort from external hardware, finger stiffness, loss of reduction, and complex regional pain syndrome are other complications [54,55,63,64,65,66,67]. Patients who receive an external fixator have reported more discomfort and reduced health-related quality of life when compared with internal fixation [53].

Some potential limitations may have influenced the results of our study: (i) its retrospective nature and the different sizes among BEF and BEF + KWs (192 vs. 72); (ii) the wide range of follow-up, from a minimum of 24 months after surgery to a maximum of 87 months; (iii) the lack of objective evaluation of range of motion in the follow-up of our patients, as well as the lack of radiographic evaluations in the study. This could have affected our clinical-functional outcomes at medium-term follow-up, as they were based only on the subjectivity of the patients, without a radiographic correlation. Another weakness is the lack of a control group treated by ORIF, which would be useful to compare the results of our technique. Finally, it is necessary to underline that the AO fracture classification used for our analysis was based on standard radiographs, as computed tomography was performed only in the cases of intraarticular injuries with multiple fragments, according to our institutional protocol.

Nevertheless, to the best of our knowledge, this is the first single-centre study reporting functional clinical outcomes of DRFs at medium-term follow-up and including beta multivariate regression analysis on a large patient cohort compared to previous published studies on the same topic [12,46,68,69,70]. Further, all patients enrolled were operated on by the same trauma surgeons and followed according to a standardised institutional post-operative protocol, reducing confounding bias. Importantly, the functional limitations of treated wrists were evaluated using two validated questionnaires whose reliability, validity, and specificity have been confirmed by several studies [71]: the PRWHE Score, a specific tool only for DRFs assessment, and the QuickDASH, a widespread method for upper limb evaluation.

Future randomised controlled clinical trials comparing the BEF procedure with optional KWs to other operative methods are necessary to better define optimal indications for the treatment of unstable DRFs in both young and elderly patients and provide further useful information in relation to fracture pattern.

## 5. Conclusions

The medium-term functional-clinical outcomes of this retrospective study and their beta multivariate analysis suggest that the use of BEF with optional KWs for the treatment of unstable DRFs can be indicated in both young and elderly patients of any gender, independent of limb side and fracture pattern. Nevertheless, as the best functional results were achieved in the elderly when KWs were added to stabilise and maintain the fracture reduction, in particular of the dominant side, the combination of BEF and KWs seems to be mainly indicated for the treatment of DRF, also complex, in the elderly population.

## Figures and Tables

**Table 1:** Overall cohort characteristics.

Variable	Patients (*n* = 269)
Age	65.55 ± 15.27
Sex	
Female	202 (75.1%)
Male	67 (24.9%)
PRWHE	12.18 ± 15.75
QuickDASH	13.13 ± 15.86
Side	
Dominant	106 (39.4%)
Non-dominant	163 (60.6%)
AO classification (23)	
A2	48 (17.8%)
A3	39 (14.5%)
B1	2 (0.7%)
B3	1 (0.4%)
C1	50 (18.6%)
C2	82 (30.5%)
C3	47 (17.5%)

**Table 2:** Comparison between BEF and BEF + KWs groups.

Variable	BEF (194 Patients)	BEF + KWs (75 Patients)	*p*-Value
Age	65.83 ± 15.24	64.81 ± 15.44	0.59
Sex			0.57
Female	148 (76.3%)	54 (72%)	
Male	46 (23.7%)	21 (28%)	
PRWHE	14.59 ± 17.04	5.88 ± 9.20	<0.001
QuickDASH	15.30 ± 17.10	7.50 ± 10.16	<0.001
Side			0.57
Dominant	79 (40.7%)	27 (36%)	
Non-dominant	115 (59.3%)	48 (64%)	
AO classification (23)			0.007
A2	41 (21.1%)	7 (9.3%)	
A3	30 (15.5%)	9 (12.0%)	
B1	0 (0%)	2 (2.7%)	
B3	1 (0.5%)	0 (0%)	
C1	40 (20.6%)	10 (13.3%)	
C2	53 (27.3%)	29 (38.7%)	
C3	29 (14.9%)	18 (24.0%)	

**Table 3:** Beta multivariate regression analysis of the impact of the different variables on PRWHE score.

Variable	Estimate	Standard Error	Z Value	*p*-Value
Intercept	−0.377	0.436	−0.866	0.386
pp	−0.976	1.739	−0.561	0.575
BEF + KWs	−0.385	0.165	−2.334	0.020
Dominant side	0.275	0.133	2.065	0.039
Age	−0.018	0.004	−4.004	<0.001
23-A3	−0.310	0.254	−1.224	0.220
23-B	−0.173	0.955	−0.181	0.856
23-C1	−0.002	0.214	−0.010	0.992
23-C2	0.006	0.386	0.017	0.986
23-C3	0.182	0.439	0.416	0.677
BEF + KWs and dominant side	−0.410	0.266	−1.545	0.122
Phi coefficients				
Intercept	0.186	0439	0.425	0.671
Age	0.014	0.006	2.551	0.011
pp	2.168	0.888	2.441	0.015

**Table 4:** Beta multivariate regression analysis of the impact of the different variables on QuickDASH score.

Variable	Estimate	Standard Error	Z Value	*p*-Value
Intercept	−0.378	0.433	−0.874	0.382
pp	0.211	1.735	0.122	0.903
BEF + KWs	−0.249	0.165	−1.516	0.129
Dominant side	0.354	0.133	2.664	0.008
Age	−0.020	0.004	−4.544	<0.001
23-A3	−0.362	0.251	−1.447	0.148
23-B	−0.616	0.960	−0.641	0.521
23-C1	−0.156	0.211	−0.740	0.459
23-C2	−0.263	0.385	−0.682	0.495
23-C3	−0.120	0.438	−0.273	0.785
BEF + KWs and dominant side	−0.468	0.264	−1.775	0.076
Phi coefficients				
Intercept	−0.012	0.430	−0.029	0.977
Age	1.546	0.876	1.764	0.078
pp	0.020	0.006	3.586	<0.001

## Data Availability

The dataset supporting the conclusions of this review is available upon request to the corresponding author.
